# Bridging the Gap: Exploring the Unique Transition From Home, School or College Into University

**DOI:** 10.3389/fpubh.2021.634285

**Published:** 2021-03-17

**Authors:** Joanne Deborah Worsley, Paula Harrison, Rhiannon Corcoran

**Affiliations:** ^1^Department of Primary Care and Mental Health, University of Liverpool, Liverpool, United Kingdom; ^2^Student Administration and Support, University of Liverpool, Liverpool, United Kingdom

**Keywords:** first-year student experience, transition period, loneliness, sense of belonging, friendships and relationships, well-being

## Abstract

Although previous research has shown that psychological distress increases on entering university, little is known about first-year student perspectives on the stressors in university environments, and what measures might better support student mental health and well-being during the transition period. The present research seeks to bridge this gap by exploring the unique transition period from home, school or college into university in order to identify aspects of the university experience (teaching, curriculum, support services, accommodation) that could be adapted to better support student mental health and well-being. Eight focus groups were conducted across two higher education institutions in North West England. Focus group data were thematically analyzed. Four overarching themes were identified: “*Tackling multiple challenges*”; “*The preparatory role of schools and colleges*”; “*University staff and services: Feeling supported/unsupported*”; and “*Friendships*.” Although pressure to perform academically whilst fulfilling the stereotypical student life is keenly felt during the transition period, many students conceal their struggles from family and friends. Living beyond their familiar support structures often leaves students in need of compassionate humans to turn to for support or advice, especially as many keep their struggles hidden. Large-group teaching settings stifle social connection and leave students feeling lonely. Loneliness was also experienced within accommodation environments. Providing increased opportunities for connection within living and learning environments would enable young people to build and strengthen relationships with fellow students and staff. As supportive environments foster a sense of belonging and community, universities should ensure that students feel supported by staff and peers whilst they acclimatize to multiple new challenges.

## Introduction

While starting university is exciting and liberating (1), the transition period can be stressful, especially for those who are relocating (2). In Britain, over 80 per cent of full-time students leave their family home to attend university (3). The transition to university coincides with a critical developmental period during which young people leave their family home whilst their brain is undergoing accelerated growth and shows heightened sensitivity to stress (4). There are a number of unique stressors associated with moving from home into shared accommodation such as living with strangers, developing independence, and managing domestic commitments (5). Many students are also required to work part-time alongside their studies to support themselves, and this often exacerbates stress (6). Indeed, psychological distress increases on entering university (7, 8). Many students who drop out of university do so during the first few weeks of term (9), with well-being concerns being the most commonly cited reason (10). On the other hand, positive transition experiences enable students to cultivate a sense of belonging, which can enhance feelings of well-being and lead to increased academic success (1, 9).

Student preparation is one factor that determines whether an individual experiences the transition as positive or negative. Indeed, the opportunity to cultivate social and navigational capital enables young people to settle quickly into their new environment (11). For example, students who completed a pre-entry programme reported higher academic self-efficacy and satisfaction than traditional route students within the first few weeks of their academic study (11).

Students entering higher education (HE) become responsible for their own learning and achievement (12, 13). This increased responsibility and the need to manage time across multiple demands can lead to increased levels of stress and anxiety (14). As students' expectations diverge from the reality of their first-year at university, their ability to adapt to their new learning environment can be hampered (15, 16). While students are expected to become independent learners during the transition period, many are unaware that this is a key requirement for success and report not possessing the necessary skills for effective independent study (16, 17). Feeling unprepared for independent learning is cited as a cause of withdrawal from studies (18).

Crabtree, Roberts and Tyler (19) interviewed college tutors and university lecturers to explore the similarities and differences in learning environments, with a particular focus on teaching styles. Whilst the primary emphasis for college tutors was on student success and achievement, university teachers placed more emphasis on motivating students to develop an interest or enthusiasm for their subject and expected students to take personal responsibility for their learning (19). According to a recent report, college teachers express concerns about the transition to independent learning (2). More specifically, as students are often “spoon-fed” information to help them achieve high grades in schools and colleges, teachers perceive self-directed learning in HE as a marked change from earlier education (2). College teachers also express concerns regarding levels of support in HE institutions, fearing that students may “slip off the radar” if they are struggling academically as university staff often have less capacity to spot signs of distress (2). Compared to school or college, university students receive less support and often experience difficulties in getting to know their academic tutors due to large-group teaching settings (20).

In line with this, students also experience difficulties in getting to know their course peers (20). Although course peers were found to be an important source of support, students' living arrangements were found to be central to the process of making compatible friends (21). Wilcox et al. found that friends in halls provide direct emotional support, equivalent to family relationships. This is especially important considering that, for many students, this is likely to be the first time they have lived away from home. However, as building meaningful relationships with others takes time, students often feel lonely during the transition period (5). According to a recent report, forming new friendships at university can be stressful, and many young people experience pressure to establish and fit in with a new group of friends (22). Indeed, a gap exists between applicants' expectations of a busy and fulfilling social life at university, and the reality that making friends cannot be guaranteed, especially with those you live with (23). Jopling and Valtorta (24) found that students were spending on average 4.2 h per day alone in their room, and over a third of those who reported spending longer than 4 h alone also reported not having friends to spend time with. Many students expect to interact more regularly with others at university more than school, and often students are disappointed when this does not happen (22). According to Neves and Hillman (25), a quarter of students who rated their university experience as worse than expected claimed that this was due to limited interaction with other students.

As the transition from home to halls engenders significant changes in young people's social networks and the primary support system often shifts away from family and high-school friends (26, 27), it is important to ensure that universities provide supportive environments (28). Although the quest to develop supportive networks is an integral part of the first-year student experience, developing new support networks may be particularly important for those who live in halls of residence as they are physically and emotionally separated from family and high-school friends. The current research aims to provide a rich insight into the resident student experience of the unique transition period through qualitative means with a particular focus on the transition to independent living. By exploring factors that facilitate or hinder a successful transition, we aimed to identify aspects of the university experience (teaching, curriculum, support services, accommodation) that could be adapted to better support students during the transitional phase.

## Methods

### Ethical Approval

Ethical approval was received from the University's Health and Life Sciences Research Ethics Committee (5762). The participants provided their written consent to participate in this study.

### Participants

A total of eight focus groups were conducted across two higher education institutions in North West England including a Russell group and a post 92 institution, namely the University of Liverpool (UoL) and Liverpool John Moores University (LJMU). Students studying at UoL reside in university-owned accommodation (UOA) whereas students studying at LJMU reside in privately owned accommodation (POA). Participants were recruited to participate in the study via bulk emails and flyers. In total, 38 first year students participated in the research, and were remunerated for their time. Thirty-three females and five males were engaged in the eight focus groups with group size ranging from four to five. The majority of participants were between 18 and 19 years old. In the context of some Research Ethics Committee concerns regarding assurance of confidentiality of those involved in these focus groups where matters to do with well-being were discussed, the authors chose not to gather detailed demographic information that could identify students.

### Focus Groups

Focus groups followed a semi-structured set of questions. Within focus groups, most students answered each question although some contributed less to the overall discussion. Examples of topics covered included adapting to university life; expectations about what it would be like; challenges during the transition; and factors that helped or hindered the process. The focus group interview schedule can be found in Supplementary File 1.

Each focus group was conducted by the first author. Data collection was carried out during November and December 2019, which corresponded to semester 1 of the first year at university. Focus groups were conducted in students' halls of residence so the environment was familiar to participants. This ensured that there was an appropriate member of staff (e.g., a residential adviser) to draw on if required. Each focus group lasted 1 h and each discussion was recorded using a Dictaphone. Data were anonymised at the point of transcription. All transcription was undertaken by the first author to allow maximum data immersion.

### Analysis

Focus group data were analyzed using thematic analysis by the first author who was trained in qualitative methods. Thematic analysis is a flexible qualitative method that aims to identify, analyse, and report recurrent themes in data. The five steps for conducting thematic analysis as outlined by Braun and Clarke (29) were followed. Initial and repeated viewing of the transcripts was undertaken, considering both contextual and reflective notes. Although there were some pre-determined areas the researcher wanted to explore, line by line coding derived from a largely inductive approach ensured that data were not overlooked. Although line by line coding was undertaken by the first author, research meetings were held throughout the coding process to examine emerging impressions of the data. The initial themes captured by coding were refined during discussions with another member of the research team to produce the final themes and subthemes reported below.

## Results

Four overarching themes were identified from the data: “*Tackling multiple challenges*”; “*The preparatory role of schools and colleges*”; “*University staff: Feeling supported/unsupported*”; and “*Friendships*.” A number of subthemes were identified in relation to each overarching theme (see Table 1).

**Table 1:** Overarching themes and subthemes.

**Themes**	***Subthemes***
Tackling multiple challenges	*Complex life transition*
	*Anticipation versus reality*
	*From a class of 30 to a cohort of 300: From spoon feeding to holding your own*
	*From home to halls*
	*Financial pressures*
The preparatory role of schools and colleges	
University staff: Feeling supported/unsupported	*Academic staff Accommodation staff*
	*Support services staff*
Friendships	*Forming friendships in a lecture hall: An impossible task?*
	*Friendships in flats*

### Theme 1: Tackling Multiple Challenges

#### Complex Life Transition

Although young people found starting university exciting and liberating, certain aspects, such as living away from home, were found to be stressful:

“*Exciting and like liberating. You were more independent from your family so I suppose it made me feel free in a way. I think over time you sort of feel stressed because some of us are from a different country, like I'm from Ireland, so it's hard not seeing your family for a few months*” (POA FG3 p1).

“*I found it exciting but also being from Northern Ireland and being away from home it's hard. I wanted to be more independent and free which is why I chose to go overseas and not stay back home because it is more liberating to able to be yourself and make your own choices*” (POA FG3 p4).

For the majority of students, this was the first time they had lived away from home, and sharing a living space with unfamiliar people was difficult:

“*Up until university I had never been away from my family for more than three days*” (POA FG1 p5).

“*It was a bit hard at first because I'm used to walking around like seeing your family in the house and you walk and you are just seeing people who you've only just met*” (UOA FG4 p1).

As starting university involves several periods of transition, many young people struggled to navigate multiple new environments:

“*We are all dealing with a lot of stuff at once you've got your accommodation, you've got your course starting, meeting friends etc. I think a lot of people are well fragile might not be the right word but they are kind of in that stage were they might be struggling a little bit*” (UOA FG3 p5).

“*It is difficult though because there are so many factors. It's not just is this the right course for me, it's is this the right city? Is this the right accommodation? Is this the right time for me to progress my educational career? There is a lot you have to consider*” (POA FG4 p3).

Thus, for many of the resident students involved moving away from home was challenging because of the need to adjust quickly to multiple new educational, social and physical environments.

#### Anticipation vs. Reality

Students' expectations often diverge from the reality of their first few months at university, and this can leave them feeling disappointed:

“*You have these expectations about what it's going to be like and then it's different, like social stuff. I get on with all my flatmates but it's just different to how everyone said it would be or you have this massive expectation of university being this life changing experience and sometimes you get here and it's just university*” (POA FG3 2).

“*Before you go to uni, people make it out as though it's going to be the most amazing experience and everyday is going to be amazing, everybody is going to be so friendly but you just have to be more realistic with yourself and accept that it's just life and it's not amazing all of the time*” (UOA FG2 p4).

More specifically, many students were expecting to experience the “best years” of their life at university:

“*I do think there is almost like a pressure that uni is going to the best years of your life, you'll love it and I didn't love it straight away*” (POA FG4 P4).

“*In the first few weeks I found it really tough because I was like why am I not having the bestest time in the world when I've been told I should be*” (UOA FG1 p2).

It is also common for students to expect to form lifelong friendships, believing that cultivating these friendships will be an easy process:

“*It's very much like you are going to find your ‘friends for life' with your flat and things like that and when it's not like that it's a bit disappointing*” (UOA FG4 p1).

“*Everyone says you will be fine making friends at uni but it's actually hard*” (UOA FG1 p1).

Students described their expectations of university as a positive “life changing experience” and being fun-filled with new friends. However, this was not the reality for most. Thus, the mismatch between the idealized image of the university experience and the lived reality was commonly heard.

#### A Class of 30 to a Cohort of 300: From Spoon-Feeding to Holding Your Own

As students in HE become responsible for their own learning, many found this to be a marked change from earlier education. In particular, young people are often “spoon-fed” information in order to achieve at school or college whereas studying at university requires greater autonomy:

“*I think with school and the way it is structured, you are spoon-fed everything from GCSEs then to A-levels and it's all part of a natural progression. Then you come to uni and it's like not only are you in a new city, you've also got new people in a new environment and then a new teaching style and it is a whole new structure of education”* (UOA FG1 p2).

“*I think it's completely different to A-levels because I've been told that I've got two essays to do and we've literally just been given our questions and they have just said do this essay and at A-level they just gave us more structure and guidance. Then you just get thrown into lectures and you're taking your own notes and if you miss one it's your own responsibility. You are more responsible for your own learning*” (UOA FG1 p1).

Compared to earlier education where young people are taught in small groups of around 20–30, students in HE are often taught in large group settings:

“*It's a very different kind of learning in terms of uni itself so it's not as one on one because there are almost 300 of us. There is no direct help you've got to put the effort in yourself* ” (POA FG2 p2).

“*I wasn't expecting the lectures to have 550 people in a lecture trying to learn everything and know everything by yourself because I did a btec and everything was pretty much just given to me. That's what they don't tell you that at university how much you've got to do yourself* ” (UOA FG3 p3).

Young people perceived school as a familiar environment where they received personalized support from their teachers. In contrast, students found that there was less personalized support on offer at university, and often felt as though they had become just “another face in the crowd” (UOA FG2 p4):

“*I feel education-wise, study-wise, coming from school to uni is whole change. In school, you are in one classroom with 30*–*40 people so the teacher knows each and every student. If any student has some problem they can just approach the teacher but in uni it is a lecture room filled with 150*–*200 people*” (UOA FG3 p4).

“*You lose your identity a bit in university. I think everyone does just because of the sheer number of people. You become just another person rather than having that whole life around you like you would have done when you were at school*” (UOA FG3 p5).

Some students struggled with their work and/or workload, especially those who were undertaking placements alongside their academic studies:

“*He told me a few problems he couldn't do and the course was a little difficult. He's actually smart but he felt left out. It was more his anxiety of being less than other people*” (UOA FG3 p4).

“*My course is a lot different to what I thought it would be. It's a lot harder and I can't handle the work which I wasn't expecting because at college I'd always do it. I do a 60-hour week in the ambulance and then my lecturers put something up about the nervous system that they want you to learn by yourself. I think a lot of it is that you have to learn by yourself and when you don't know what you're learning it's quite hard to do that*” (POA F2 p4).

“*We don't have to go to lectures as we just get sent them but even so they give us more hours of lectures than you could even fit into those days so it isn't like being in 9*–*5*” (UOA FG2 p5).

In summary, following the marked change from earlier education, students expressed genuine concern regarding their ability to carry out educational tasks such as completing work, acquiring knowledge from online lecture content, and keeping up with the standards of academic excellence set by other students.

#### From Home to Halls

There are a number of unique challenges associated with moving from home into shared accommodation such as developing independence and managing domestic commitments. Many young people referred to a reliance on parents:

“*I'm only 18 and you are just thrown from a pit of being at home with your parents 24/7 sorting everything out to being by yourself, to doing everything by yourself and it is stressful trying to adapt to that situation*” (POA FG3 p4).

“*When you were at home you never thought you would have so much responsibility because when you are at home you get fed three times a day, your mum does your washing, literally everything, you come home and she's changed your bed*” (POA FG4 p2).

Further illustrating the dynamic of interpersonal dependence, certain aspects of independent living, such as cooking and cleaning, were highlighted as challenging:

“*I'm used to coming home to a plate of food to thinking have I got the meat? Did I take it out of the freezer? Everyday I forget to take the meat out of the freezer and I end up going to the takeaway*” (POA FG4 p1).

“*The amount of times I've literally just been sat in my room and I've just forgotten to make my own tea and then it's got to 9 o'clock and I'm like oh I should have done that*” (POA FG1 p4).

Some students acknowledged that their diet had worsened upon starting university, and this had an impact on their physical and mental health:

“*I don't buy fruit for myself. I'm used to my mum buying it and telling me to eat it and then I realized I'm tired all the time and my diet is absolutely awful”* (UOA FG4 p2).

“*At one time I didn't want to speak to anyone. I didn't want to go out. I just wanted to be alone in the room. I had to force myself to go and socialize which was really hard for me but it was all down to what I was putting in my body*” (POA FG3 p4).

As parental figures often take control of the household duties and chores, some students found it difficult to share a space in a co-operative way:

“*I have really bad OCD and they don't understand that if you leave pots on the side it's going to be used and if you leave it dirty it really stresses me out*” (POA FG4 p1).

“*If you've got that thing were you want the dishes to be washed straight away and another person doesn't, it then becomes that's annoying me. If I try to deal with it and say look can you please wash it, and they don't like it, it then starts an argument*” (UOA FG3 p3).

Living in student accommodation alongside strangers often leaves young people feeling lonely and homesick, especially if they feel they do not fit the mold:

“*I got homesick because you are away from your family and you are basically living with people you've never met in your life so it's quite odd*” (POA FG1 p2).

“*I got here and I was like I just want to be at home because I was just so lonely. I don't really go out and I thought there would be more to do if you didn't go out*” (POA FG4 p3).

This subtheme illustrated the adjustment associated with moving from home to halls that resident students go through. For the majority of students, this will be the first time they have lived away from home, and they have to start managing many aspects of life independently. Feeling lonely and homesick is a common experience.

#### Financial Pressures

Many students struggled financially. Budgeting was often cited as a cause of worry:

“*There is also the finance stress of it as well. That's a massive thing and budgeting because it's like I want this thing but I realistically shouldn't do that*” (POA FG2 P2).

“*I think budgeting as well. Say I have 50 pounds per week to spend on everything so it's knowing how much of that money to spend on food for the week and how much to spend say if I went on a night out. I find that quite hard*” (POA FG1 p2).

Financial concerns were heightened for those undertaking placements alongside academic studies:

“*I'm doing nursing and it's full-time so I can't get a job so then I'm having to ask my mum for money for trains to get to placements and it's just very stressful. It causes a lot of stress really*” (POA FG1 p2).

“*I think for us with nursing we have to pay £4 for the train ticket. £4 a day when you are on placement four times a week, that takes up quite a lot of your £50 budget and you've got to remember that every month you have your phone bill which is £20*” (POA FG1 p4).

Accommodation costs take an increasingly large portion of young people's student loans. In fact, some students highlighted that accommodation costs were either equivalent to, or greater than, their student loan:

“*My loan didn't cover my accommodation. My parents last month had to pay an extra 600 pounds toward the accommodation and have to give me money weekly”* (POA FG1 p2).

“*I think I've managed to do my finances well because my cost of rent and the amount I get from student loan is basically like the same so I can disregard them both*” (POA FG1 p5).

As a consequence, many students worked part-time alongside their studies to support their living situation; however, this often exacerbated stress:

“*If you can't afford things like you've got to balance if you can afford food or going out and which is the better option. I've got two jobs as well as uni because I've got to pay for my life basically so it's like added pressure*” (UOA FG2 p1).

“*I have found trying to juggle my money hard so I have had to work a little bit as well just to make sure I've got enough money but then when you are working you are not concentrating on your degree as well so I have found it hard to balance them both*” (POA FG3 p3).

Pulling this together, it seems that many students felt that they lacked budgeting skills. Financial pressures led to students working alongside their studies, which was perceived to exacerbate stress. Parents need to provide financial support added to the sense of pressure with many students taking part-time jobs as well as relying on parental support.

### Theme 2: The Preparatory Role of Schools/Colleges

As many students struggled to acclimatize to their new living environment, it was felt that schools and colleges could prepare students for independent living by embedding preparatory life skills workshops into curricula:

“*Sometimes I think it's not the actual uni that's stressful, it's more the living and budgeting and living with other people and you are not really told about any of that. Maybe teach us more about budgeting and life skills*” (POA FG2 p3).

“*We had no support on money or even like cooking meals. Even though it's like the little things, it is what makes your experience living in halls so much different”* (POA FG1 p4).

Although schools and colleges help young people with the application process, young people would appreciate assistance in preparing them for self-directed learning and the independence that ensues:

“*My college did really help with the academic side so ‘you need to do this to go to uni'. UCAS forms they helped us so much and applying and looking but then it's the actual transition from going from college 9-4 to being in uni and being totally independent with your studies and your life in general*” (POA FG1 p4).

One way by which school or college teachers could prepare young people for higher education is by treating them as adults and giving them additional responsibilities:

“*I think they could treat you more like an adult in school because I think they continue to treat you like a child*” (UOA FG3 p3).

“*One of the things with sixth form is that the teachers say oh we are going to treat you like adults now and then they don't*” (UOA FG2 p5).

Taken together, as schools and colleges focus only on the application process, students felt ill-prepared for living at university. The data suggest that students would welcome the opportunity to attend workshops in FE settings focusing on cooking, finances, budgeting, and independent learning and living to ensure that they are adequately prepared for the transition to university.

### Theme 3: University Staff and Services: Feeling Supported/Unsupported

#### The Role of Academic Staff

Some students felt supported by their lecturers in their new learning environment:

“*I do fashion communication so my course is really small so my lecturers know me really well which is a good thing. I can email my lecturer and be like ‘oh I'm having a meltdown' and he would be like ‘right don't panic, come and see me at this time'. He knows who I am. That's the thing I really like*” (POA FG4 p3).

“*At uni they do treat you like an adult but they treat you like an adult who is fragile and who needs to be taken care of which is exactly what I am*” (UOA FG2 p5).

However, other students did not feel adequately supported, and were often left feeling as though they were “another face in the crowd”:

“*I did expect to have some more support from my lecturers. I think it's quite easy to just slip through the net with it and have no idea what's going on when everyone else does. It is quite hard to ask for help especially when you're in front of so many people*” (POA FG2 p2).

“*I thought we would get more support from the uni than we do because I feel like we get no support at all not with my health and stuff but the work as everything is online and the teachers make you feel like you're being an inconvenience if you email them so they do these online discussion boards but I don't like them. I really don't*” (POA FG2 p3).

“*We got told our academic advisers and things like that and I had in the back of my mind I've got this academic adviser if I am super stressed it's fine but then nothing has happened with them and I haven't seen them and I couldn't remember who they were. I was expecting a meeting*” (UOA FG4 p3).

To summarize this section, students undertaking courses that have a small cohort size feel able to approach their lecturers and regard them a source of assistance when they are experiencing difficulties. By contrast, those who are part of a larger cohort of students do not perceive the support to be adequate, and identified problems around lack of approachability and availability.

#### The Role of Accommodation Staff

Within shared accommodation, young people value staff who are friendly and approachable, and they feel valued when operational teams organize events:

“*I think it's good that you come in and there's always going to be someone on reception and they're so nice and they are so friendly. There are a couple of young people so it's quite good as you can go and say something that you might not want to say it to someone that's like your parents age*” (POA FG4 p3).

“*Staff-wise everyone is lovely and friendly and they want to be there to support you and do little things like the Halloween party they did for us. It makes you feel as though they do really care about you. You are not just a flat number, you are a person because they are doing it for you*” (POA FG3 p4).

In university-owned accommodation, residential advisers (RA) live alongside students in order to offer support. There were, however, inconsistencies, as some students felt supported by their RA whereas others did not:

“*I've got [name of RA] and she's so nice. I think it was yesterday or this morning, she came in and just said how are you all getting on kind of thing. She literally just came in for a 10 minute chat like is everything ok? How are you getting on? So I do feel supported by the RA*” (UOA FG2 p5).

“*I've only seen the residential adviser [once]. Are they just there to stop fires or are they there for support?. He hasn't dropped in to say I'm still here if anybody wants to talk to me. I don't even know what room he is in so if I did need to find him I would be lost*” (UOA FG4 p2).

In light of this, it would be beneficial for all RAs to be proactive in getting to know the students living on their floor in order to enable them to feel comfortable approaching their RA in a crisis:

“*Even if they just drop by every two weeks for 5 minutes, that would be more than what we've had*” (UOA FG4 p2).

“*People can present themselves in a certain way and then go back to their room and be depressed, anxious and they don't want to put on the front as being the depressed person in the flat so they hide it very much because they want to look and fit in with everyone else and not be the person who causes problems by not feeling 100% so I feel like definitely having services share with accommodation is definitely be beneficial and just having little prompts to make sure people are ok can make the world of difference for people*” (UOA FG4 p1).

Taken together, operational teams and residential advisers appear to be a source of support for resident students. Many students discussed the importance of having friendly and caring staff who they feel able to approach and confide in.

#### Support Services

Mental health and well-being support services are widely advertised across university campuses, and students found this to be beneficial:

“*I think from what I've seen the university has been really on top of mental health issues. There is a lot of information out there and they do things on Instagram and Facebook and it is really pushed so honestly from my perspective the university does do a lot from a mental health perspective, especially for new students*” (UOA FG4 p5).

“*When you are walking through uni there are posters everywhere and billboards even, and the big cardboard stands. Everyone makes sure that not only is there support, it's in your face here is the support so people walking past, you don't have to walk up to someone. The information is put up there for you to see which is quite good*” (POA FG3 p1).

On World Mental Health Day, UoL support services had a stall in university square to advertise their mental health support offer, and students valued this informal approach:

“*There was the World Mental Health Day and there was a stand and the free coffee was an incentive to go over and talk to people. You could just go up and talk to people so I think doing things like that where it's not an organized event so someone might be having a really bad day and seeing that stand walking past, saw there was free coffee and just walked up. Just something that is really casual because someone would feel more open to talk*” (UOA FG4 p3).

Students discussed the importance of casual support offers, and would welcome the opportunity to have “mental health check-ups”:

“*You have check ups for physical health, why can't you have check up for mental health?*” (UOA FG4 p1).

“*You see so many people saying we need to talk about mental health and people get scared of it so it almost has the opposite effect so if it was just casual it would be normal*” (UOA FG4 p2).

Some students suggested ways in which university support services could be improved. At present, there is an over-reliance on students to communicate causes of stress. However, young people are often reticent and prefer to keep their struggles hidden:

“*They have mental health and disability drop-ins sessions but it is very much up to the individual to put the effort in to go and attend those when there's not as much online support than there is the face to face support*” (UOA FG3 p1).

“*I know you can still use ChildLine up until 19, but if there was something that was university-focused that would be quite beneficial. So if you were in your room isolating yourself, that would be very beneficial in my opinion because you wouldn't have to leave your room. You don't have to fear speaking to anyone and what you might need to release, in terms of what has built up inside you could really benefit from typing it, writing it*” (UOA FG4 p4).

Last, some students face the challenge of discontinuity of support on arriving on campus as the transition between home and university disrupts the established therapeutic relationship with their clinician. Due to high demand for psychological therapies, it is common for students to join a waiting list at university. Thus, it would be beneficial for universities to support vulnerable young people whilst they await therapy:

“*I know you instantly get referred for support off the university but sometimes just more of a extra bit of help to say you've been having therapy back home up until you move to university and then you suddenly have that striped away from you like more of a transition into a different sort of help rather than just going into not having any help and having to wait a significant amount of time if you are used to a certain way of being seen by someone*” (UOA FG4 p1).

“*My mum was very weary because we know how hard it is to get a therapist or anything across the country at like your age and waiting lists are ridiculous so we were very much like you don't want to have that in the back of your mind that you're on a waiting list at uni. You almost just end up being an object on the waiting lists like you don't really feel like you are a priority and I think that would have stressed me out more if I tried to start it here”* (UOA FG4 p3).

Data falling under this subtheme illustrated that some students expected poor care from the outset because of long waiting lists. Others articulated the importance of having an online support service staffed by people who are familiar with university-specific issues.

### Theme 4: Friendships

#### Forming Friendships in a Lecture Hall: An Impossible Task?

Some students felt integrated within their school or department, and a sense of belonging was often derived through feeling connected with others on their course:

“*I do feel like I belong as a Liverpool physio[therapy] student because I've found people I get on with and we are together all the time*” (UOA FG2 p5).

“*I feel part of the Classics department. I think we are very close as a department*” (UOA FG2 p2).

Attending a course induction week or reception was highlighted as an important way to form friendships with course peers:

“*We had the reception at Garston museum because we are doing Classics. We met there and we found some other people there so that's how we found friends really and everyone in Classics are nerds so we all have the same interests*” (UOA FG2 p2).

“*The people I met on my course in the induction week, I'm still friends with them now and I feel without that I would have had a different experience*” (UOA FG4 p4).

Forming friendships with course peers is important as peers provide a source of emotional and practical support:

“*There are 70 of us and you go out in the ambulances and you can see anything so you use everyone as a support mechanism*” (POA FG2 p2).

“*Support system. We do the same course so if one of us needs help we can help the other person even if it is just proof reading something for someone that is helpful*” (POA FG2 p4).

Some people experienced difficulties forming friends with their course peers, and many cited large-group teaching settings as a barrier:

“*I also think it's quite hard to find friends on your course because when you are in lectures you can't really speak to anyone and then in seminars not really either. I do Psychology and there are 300 people there so I literally can't speak to anyone*” (UOA FG2 p1).

“*I haven't made that many friends on my course, especially in the lectures, everyone sits in a different place each time so you never really see someone for long enough to start making friends*” (POA FG2 p3).

In fact, large-group teaching settings stifle social connection and leave students feeling isolated:

“*You still kind of feel by yourself even though you are in a lecture hall with 300 other people. You still feel like just by yourself because you don't know anyone. You don't have anyone to talk to*” (POA FG1 p4).

Students acknowledged that small teaching groups, such as seminars and tutorials, are useful in aiding the process of friendship development as these settings facilitate student-student interactions:

“*It's more in your tutorials that you can actually speak to someone because it's a smaller group. Even then it's like you are just speaking to someone for an hour and then you'll go your separate ways until next week*” (POA FG2 p3).

“*With our course we don't have seminars we only have lectures literally just lectures so seminars where you go into a group and talk and stuff we don't have any of that side so we literally just sit there, have someone talking at us and then go. We don't have any group seminars where you sit there talking to other people*” (POA FG1 p2).

In summary this subtheme illustrated that the course structure can facilitate or impede the pursuit of supportive friendships. Students desire more opportunities for connection using small group teaching and learning methods as simply being on campus attending lectures does not appear to be enough to reduce feelings of loneliness.

#### Friendships in Flats: Throwing People Together

Feeling integrated within shared accommodation is also important during the transition period. Some young people gelled with their flatmates easily, and this had a positive impact on their experience:

“*In this accommodation, I have gelled with a lot of people to the point of hobbies and interests*” (POA FG3 p4).

“*I didn't find it as hard as I thought it was going to be but I think that's because the people I met in my very first week, my flatmates, are really lovely*” (UOA FG4 p4).

Flatmates are able to provide a source of emotional and instrumental support, which is particularly important as young people's familiar support network becomes remote when they move to university:

“*I remember one night I was properly upset here and all I wanted was my mum and it was just hard because a phone call is not the same as seeing in person but yet again if you do have good flatmates it helps to be honest so you can talk to them*” (POA FG3 p4).

“*I've found it really hard being away from my family. I think it depends on who your flatmates are as well because I found it a lot easier to talk to my flatmates as opposed to if I didn't get on with them so that's made it easier*” (POA FG3 3).

As some students' support needs are adequately met by their flatmates, over time this source of support begins to replace their reliance on their familial support system. However, others struggled to form interpersonal bonds with their flatmates due to different preferred means and methods of socializing or lack of shared interests:

“*I don't get on with my flatmates that well. We don't have similar interests or hobbies*” (UOA FG2 p3).

“*Everyone in mine is quite antisocial and quiet. I do feel quite lonely at times because they are not the people to come out and socialize with you*” (POA FG3 p4).

Despite experiencing loneliness and/or perceiving their direct social support as inadequate within their accommodation environment, some students concealed their struggles:

“*I think it can be quite isolating as well because you move away from your friends and family and then if you're not really making friends, you feel quite lonely and I think you start to panic and then you might not necessarily want to tell your parents you're feeling like that because you don't want them to worry and you don't want to stress them out and then you just end up feeling lonely by yourself* ” (POA FG2 p1).

Young people would welcome the opportunity to foster friendships with fellow students living in their accommodation:

“*As much as we are adults it would be nicer to have more support in getting to know people and stuff like making it feel more of a community because I do feel as though we have just been left to our own devices*” (POA FG1 p4).

“*Even if it was a case of your floor in your block, having like an opportunity to arrange events because then you will have more contacts on your floor and that's not necessarily (name of accommodation) wide but it will still create a sense of community if you are making more friends in your Block because then if you are having difficulties with your flatmates then you still have someone to reach out to who is relatively close to you*” (UOA FG4 p1).

Students highlighted the importance of living with similar others who are able to provide the social support that is so crucial during the transition period. Fellow residents share a mutual understanding and can provide immediate emotional support. Nevertheless, as some students struggled to form interpersonal bonds with their flatmates, loneliness was keenly felt by some.

## Discussion

The present study provides rich detail of the early first year student experience from the perspectives of those living in halls of residence. Consistent with previous research [e.g., (21)], our findings demonstrate that resident students require support whilst navigating their new academic environment, and as they acclimatize to their new living environment without their pre-existing support networks of family and friends. Although pressure to perform academically whilst fulfilling the stereotypical student life is keenly felt during the transition period, many students conceal their struggles from family and friends.

In line with previous work [e.g., (5)], there are a number of unique challenges associated with the transition. One major challenge young people encounter is developing independence. Before moving to university, young people often have one or two elders advising them on every aspect of life. Young people also display a similar pattern of reliance on teachers for help. Living outside of these familiar support structures often leaves students in need of compassionate humans to turn to for support or advice, especially as many conceal their struggles. Consistent with previous research [e.g., (20)], some students found it difficult to make friends with their course peers. Large-group teaching settings stifle social connection and leave students feeling lonely. In line with previous research [e.g., (21, 24)], loneliness was also experienced within accommodation, with some students reporting dissatisfaction with the levels of support offered within halls. As these findings illustrate the importance of the human element during the transition period, universities should ensure that first-year students feel supported by staff and peers in residential and learning environments as they acclimatize to multiple new challenges.

### Recommendations for Schools and Colleges

As the transition from school or college into university is a period of significant change, greater communication between schools, colleges, and universities would be beneficial. The data presented here suggests that a joined-up approach between schools, colleges, and universities is vital in order to achieve continuity of support. For example, perhaps HE institutions should receive more information from schools or colleges on their students' needs and any generic difficulties they have encountered. This would enable universities to appropriately support young people on arrival, and may prevent a young person from repeating details on any previous negative experiences.

As many students highlighted that schools and colleges focused only on the application process, preparing students for the transition into university may be beneficial. Workshops within FE settings that focus on cooking, finances, budgeting, and independent learning and living could be delivered to ensure that young people are better prepared for the transition to independence at university. Consistent with previous research [e.g., (30)], educating prospective students on budgeting and the cost of university before arrival may prove beneficial.

### Recommendations for Academic Staff

During the transition period, academic advisers should be more proactive in getting to know their students to enable young people to feel comfortable approaching them if they are struggling with their academic studies. This could involve meeting with each student on a one-to-one basis to cultivate a trusting relationship, and to ensure they feel comfortable in their new learning environment. Consistent with previous research [e.g., (2)], it is important for academic advisers to offer more frequent pastoral support in a way that is mindful of the transition as students' feelings about their environment often impact their academic performance. This approach seems particularly advisable for courses with large numbers and lecture-based teaching methods.

To aid friendship development during the transition period, course induction events are critical. Similarly, as students desire more opportunities for social connection, particularly in large-group teaching settings, academic staff could embed icebreaker activities into lectures to encourage peer interactions. As there is a need to reduce feelings of isolation in large-group teaching settings beyond the induction period, there are clear strategies, consistent with Social Identity Theory (31), around making small changes to curricula in order to increase identification with university and the course itself. For example, within large-group teaching settings, belonging-focussed learning activities could be embedded throughout curricula, and consistent with previous research [e.g., (21)], it may be beneficial for courses to implement more group-based projects to foster connections. As tutorials and seminars further support this idea, it may be beneficial for some courses to rethink the curriculum, especially those where learning opportunities are only provided in the context of large-group teaching settings.

It would be useful to address key issues that are important to people's well-being within smaller group settings such as tutorials. For example, discussing matters such as the transition into university and the process of friendship development using light-touch facilitation would open tutorial groups up to a more natural discussion. While such emotionally live group experiences are not commonplace in HE settings, enabling students to settle into a system that is less hierarchical and more focused on wisdom and experience would be beneficial. Whilst living at home, young people draw on parental wisdom, and as emerging adults, they are still developing socio-cognitive skills. As tutors have wisdom in the area that students are working toward and also in the lived experience of the HE system, both of those knowledge bases could be drawn on within tutorials. This approach would be beneficial with regard to students' academic performance, especially in disciplines that integrate group-based assessments, as sessions addressing key matters that are important to people's well-being will enable students to develop trusting and co-operative relationships with each other. These discussions help “we-ness” to emerge as groups of people connect with each other.

In addition to this, a structured peer-mentoring programme should be considered across schools within each university department. Peer mentors, who could be third- and fourth-year university students, would perform an integral pastoral duty that could be recognized and rewarded. As young people are isolated from sources of support when they move from home to university, it would be beneficial to simulate the small family-like community within their new learning environment. Parents would be simulated through the tutor system whereas older siblings could be simulated through the peer mentoring system. While challenges exist in establishing such initiatives, peer-mentoring programmes offer valuable benefits such as the promotion of well-being and acquisition of new skills for both student participants and student facilitators (32).

### Recommendations for Accommodation Providers and Teams

Social Identity Theory (31) offers clear strategies that could be implemented within accommodation environments. To better manage the transition, accommodation teams could involve groups of students in the process of making decisions about the decoration of communal spaces in their accommodation. More specifically, students could be given the opportunity to make decisions about the plants, pictures, and layout of their lounge and dining areas. Consistent with previous research in care home settings (33), engaging with fellow students in this way may give young people a greater sense of psychological comfort and empowerment with a view to increasing social identification.

During the transition period, pastoral staff should be more proactive in getting to know the students living on their floor in order to enable young people to feel comfortable approaching them in the event of crisis. This could involve meeting with each student on a one-to-one basis to foster a trusting relationship, and to ensure that they feel comfortable in their accommodation environment. This would also enable pastoral staff to be in a position to notice deterioration in students' mental health. Pastoral staff could also meet their students as a group to introduce them to others who live near by.

As students often struggle when their experiences diverge from their romanticized expectations, it would be beneficial for universities to reconsider the marketing material that is disseminated to university applicants. Consistent with previous work [e.g., (34)], a more sensitive marketing message could help the transition as the belief that university years should be the “best time of your life” can exacerbate feelings of isolation experienced by many. Indeed, those who access pre-entry resources conveying a realistic picture of the university experience find it helpful in informing their decision about whether university is right for them (35). Educational institutions and accommodation providers should also revisit the potential implications of accommodation costs, as often students' loans do not cover the cost of their accommodation.

### Recommendations for Support Services

It may be useful for support services to engage students using a wider variety of methods. For example, first-year students could be assigned a well-being adviser and a well-being peer group. By allowing students to develop a trusting relationship with a well-being adviser, they may be more likely to disclose important information and seek support before they reach crisis point. Well-being advisers would also be well-positioned to notice deterioration in a student's mental health.

On WMHD, students valued the opportunity to interact with well-being advisers who were familiar with the challenges they were encountering. As the benefits associated with a more informal mental health support offer were acknowledged, a number of similar events could be held during the transition period and throughout the academic year. As some young people report feeling too anxious to leave their bedrooms during the transition period, it may be beneficial to develop a transition-specific virtual help offer, which could be similar to ChildLine (https://www.childline.org.uk), where young people have the option to either call or type their concerns. Indeed, for those who are vulnerable, online or phone counseling may be beneficial, especially if they are not willing to seek face-to-face support. As students are a transient population, online resources may be particularly relevant, and many students hold favorable attitudes toward online forms of support (36).

Further to this, as advocated by Universities UK (37), our findings illustrate that joined-up working between universities and NHS mental health services is urgently required. Young people in our sample expressed concerns about not receiving continuity of care at a time of significant change, which requires them to adjust to a new environment without the help of familiar support networks of parents, teachers, and school friends. When relocating, students are often unable to access NHS mental health services due to long waiting lists, which leaves them without support in a new environment. A fast-track approach should be adopted to ensure that young people with pre-existing mental health problems are able to access continuity in their care when moving from home to university. This could be from one NHS mental health service into another or from an NHS mental health service into university mental health services.

### Limitations and Future Research

Several limitations of this work should be acknowledged. As participants in this research were predominantly female and all came from two university campuses situated in a small geographic location within a North West England city, our findings may not be generalizable to the whole student population.

The findings only reflect the views of those living in shared accommodation and therefore cannot be generalized to the whole student population (e.g., commuter students). There are likely to be differences between resident students and commuter students in terms of integration with regard to both academic studies and extracurricular activities. Taking this into consideration, future investigation that elucidates the similarities and differences between resident and commuter students would be beneficial. Future research should therefore explore differences in common mental health difficulties between students who live in shared accommodation and those who live elsewhere (e.g., commuter students), and how the development of friendships and group memberships relate to this. As the findings only reflect students' perspectives, future research could explore how lecturers, residential advisers and student support services staff perceive the challenges for new university students.

There is a need to develop interventions that reduce feelings of isolation in large-group teaching settings. As our findings clearly demonstrate that students desire more opportunities for social connection particularly in large-group lectures, designing setting-based interventions that enhance belonging by embedding learning activities that encourage social connection within course content topics would be beneficial. As many efforts to enhance social connections within universities are often *ad hoc* and unevaluated (38), future research should evaluate setting-based interventions that aim to reduce loneliness, increase engagement, and improve mental health within traditional large-group teaching settings.

### Conclusion

Starting university involves several transitions such that resident students are required to tackle multiple challenges within new environments without the help of established support networks. Our findings demonstrate that academic, residential, and well-being advisers could be more proactive in supporting vulnerable young people during the transition period. In line with creating a psychologically healthy environment, providing increased opportunities for connection would enable students to build and strengthen peer relationships. With minor changes to living and learning environments, this human touch is possible, and friendly and supportive environments will foster a sense of belonging and community. Research findings like this can only have risen up the priority agenda during the current global COVID-19 pandemic. The enforced restrictions on movement, teaching and learning methods and socialization will have impacted greatly on first year students' transition to university life. When things return to normal, there will be a need to reinforce support and sense of belonging for incoming first years as well as those progressing to second and third years of undergraduate study following what has been a vastly disrupted university experience for them.

## Data Availability Statement

The datasets presented in this article are not readily available because the data collected is sensitive and could compromise the confidentiality and anonymity of the participants. Requests to access the datasets should be directed to Joanne Deborah Worsley, jworsley@liverpool.ac.uk.

## Ethics Statement

The studies involving human participants were reviewed and approved by Health and Life Sciences Research Ethics Committee, University of Liverpool. The patients/participants provided their written informed consent to participate in this study.

## Author Contributions

JW collected and analyzed the qualitative data, wrote the draft of the manuscript, and wrote the final version of the manuscript. RC and PH read, commented on, and revised the manuscript providing important intellectual input. All authors contributed to the article and approved the submitted version.

## Conflict of Interest

The authors declare that the research was conducted in the absence of any commercial or financial relationships that could be construed as a potential conflict of interest.
